# An Overall Glance of Evidence Supportive of One-Hour and Two-Hour Postload Plasma Glucose Levels as Predictors of Long-Term Cardiovascular Events

**DOI:** 10.1155/2019/6048954

**Published:** 2019-12-18

**Authors:** Baldeep K. Mann, Janpreet S. Bhandohal, Jungrak Hong

**Affiliations:** New York City Health and Hospitals/Metropolitan Hospital Center, New York, NY, USA

## Abstract

This review summarizes the vast literature describing the long-term epidemiological studies with emphasis on postprandial glucose as a stronger predictor of cardiovascular complications as compared to fasting glucose and HbA1c. Many molecular studies also supported this fact by illustrating that postchallenge hyperglycemia is associated with elevated biomarkers of systemic inflammation in the plasma and thus increasing the chances of vascular damage. Large-scale studies have proved that vascular stiffness, brachial-ankle pulse-wave velocity, carotid intima thickness, and left ventricular hypertrophy have been associated with postprandial glucose as compared to fasting glucose or glycosylated hemoglobin.

## 1. Introduction

The cardiovascular impact of elevated postprandial glucose has been studied extensively through a significant number of prospective longitudinal, cross-sectional, molecular, and experimental studies in various parts of the world. This concept of relating the postprandial glucose to coronary and cardiovascular events began as early as 1970s with some of the prospective studies at that time. The postprandial state that has been shown to be related to cardiovascular events from time to time was either after 1 hour or 2 hours of ingestion of oral glucose load also known as the oral glucose tolerance test (OGTT). This may be related to the fact that some cases of diabetes (strong risk factor for cardiovascular mortality) can be missed if only fasting blood glucose (FBG) is used in screening [1]. Only few cross-sectional and prospective studies favor glycosylated hemoglobin (HbA1c) being superior to FBG and 2-hour plasma glucose (2 hPG) for prediction of ischemic heart disease [2]. In a cross-sectional study, elevated plasma levels on admission, HbA1c, and FBG were found to have low sensitivity to detect undiagnosed diabetes in patients with acute coronary syndrome as compared to OGTT [3]. The EUROASPIRE IV (the European Action on Secondary Prevention through Intervention to Reduce Events) cross-sectional survey of 4004 subjects in 24 European countries showed that OGTT identified a largest number of previously undiagnosed diabetic patients with established coronary artery disease [4]. In a meta-analysis showing that impaired fasting glucose was significantly associated with future risk of coronary heart disease (CHD), the subgroup analyses showed that risk of CHD was only increased in the studies which enrolled participants with increased 2 hPG and not in those which excluded them [5].

## 2. Pioneer Epidemiological Studies

One of the initial investigations showing the relationship of glucose tolerance to the incidence of CHD-associated death and nonfatal myocardial infarction (MI) was two cohort studies consisting of 3,267 and 1,059 Finnish men in 1972 who were followed up over a period of 5 years and the CHD-related death was significantly related to high 1-h postload blood glucose (1 hPG) level [6]. Later, another large prospective study, the Honolulu Heart Program in Hawaii, involving a 20-year follow-up of 457 migrant Japanese patients also found 1 hPG to be positively related to MI-related mortality [7]. Also, in the Honolulu program, odds ratio for 1 hPG in patients with ankle brachial index <0.9 was 1.3 (*p* < 0.05) and found to be predictive of peripheral vascular disease 25 years later [8]. The Helsinki Businessmen prospective study in which 610 men joined a multifactorial primary prevention trial of cardiovascular diseases revealed that 1 hPG was significantly associated with total mortality along with other traditional risk factors (smoking, blood pressure, and cholesterol) after 28 years of follow-up [9]. The evidence strengthened further in the late 1990s with the Chicago Heart association Detection Project in Industry where a 22 year-long longitudinal study of 26,753 nondiabetic men and women showed that 1 hPG level is an independent risk factor for fatal CHD [10].

## 3. Subsequent Epidemiological Studies Supportive of Cardiovascular Impact of Postchallenge Hyperglycemia

**Figure 1:**
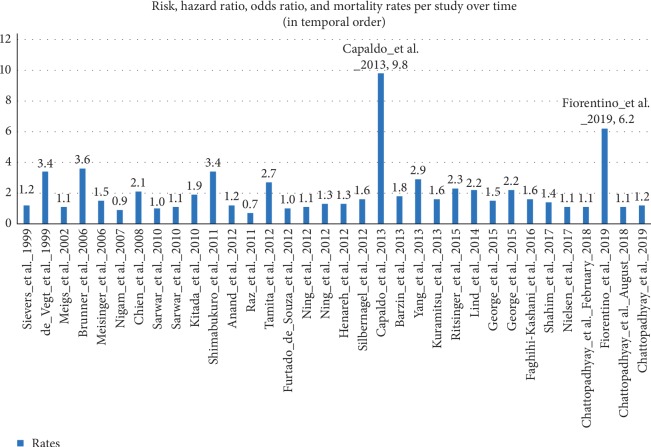
Graphical presentation of risks, hazard ratios, odds ratios, or mortality rates per study enlisted in Table 1 in temporal order.

US National Library of Medicine (Pub Med.gov) was searched with search terms “post load glucose” and “cardiovascular”; “post load glucose” and “coronary”; “post challenge hyperglycemia” and “cardiovascular”; “post challenge hyperglycemia” and “coronary”; “post prandial glucose” and “cardiovascular”; “post prandial glucose” and “coronary”; “OGTT” and “cardiovascular”; and “OGTT” or “coronary”. Various epidemiological studies were found to be conducted from 1999 to 2019 suggesting postprandial glucose as a better predictor of cardiovascular disease independent of FBG that have been enlisted in Table 1. The median follow-up in these trials was 7.2 years with a median number of patients 1425 and median rate of 1.5. Graphical presentation of the rate ratios of studies enlisted in Table 1 has been depicted in Figure 1. Epidemiological data from 20 European studies concluded that 31% of the diabetic patients with nondiabetic fasting glucose will remain underdiagnosed if only fasting glucose criteria is used and the risk profile of the subjects with impaired fasting glucose depends on 2 hPG levels [42]. Thus, the DECODE study (Diabetes Epidemiology: Collaborative analysis Of Diagnostic criteria in Europe) has emphasized on postchallenge hyperglycemia as the main determinant of risk of cardiovascular disease (CVD) in patients with diabetes [42]. A large prospective Whitehall study involving 17,869 subjects followed up over 33 years established a linear dose-response relationship between postload plasma glucose and CHD mortality risk [14].

### 3.1. Postprandial State Enhances Atherosclerosis in Arteries and Has Detrimental Effects on Myocardial Function

The epidemiological studies were also supported by experimental studies which indicated that the postprandial state may affect atherosclerosis process in the arteries, thereby increasing the coronary vascular events. In European nondiabetic population of 403 subjects, multivariate analysis showed that carotid intima-media thickness was significantly increased in the top 5^th^ quintile of 2 hPG but not the fasting glucose [43]. There was clustering of risk factors such as body mass index, waist to hip ratio, elevated triglycerides, and decreased high-density lipoprotein (HDL-cholesterol) levels in the top quintile of 2 hPG as well [43]. Another cross-sectional study of 356 participants from Pittsburgh site of the Cardiovascular Health Study measured the aortic stiffness through aortic wave pulse velocity, and after controlling age and systolic blood pressure, the strongest predictors of aortic stiffness were heart rate and 2 hPG (*p*=0.063) [44]. In the RIAD study (Risk factors in Impaired glucose tolerance for Atherosclerosis and Diabetes) intima-media thickness of common carotid arteries that was taken as measure of atherosclerosis was associated with 2 hPG even when HbA1c was within normal range [45]. This has formed a basis of many studies in 1990s like Diabetes Interventional Study, Kumamoto study, DIGAMI (Diabetes and Insulin-Glucose infusion in Acute Myocardial Infarction) study, and STOP-NIDDM (Study TO Prevent NIDDM) trial to control the postprandial hyperglycemia in order to prevent CVD [45].

In a randomized study where 61 patients with type 2 diabetes were followed up over 24 months and diastolic myocardial dysfunction (E′), intima media thickness and arterial stiffness were significantly higher in patients receiving only conventional insulin therapy (human insulin b.d.) vs. regimens receiving better postmeal glucose control with intensified conventional insulin therapy (lispro at meals and NPH bed time) and supplementary insulin therapy (regular insulin at meals) [46]. In a cross-sectional study from China involving 671 men and 603 women, it was found that the patients with impaired glucose tolerance after glucose challenge have higher brachial-ankle pulse wave velocity/arterial stiffness, are more insulin resistant, and have worse lipid profile [47]. A cross-sectional study of 767 never treated hypertensive subjects showed that 1 hPG of ≥155 mg/dL is a major determinant left ventricular mass index and hence left ventricular hypertrophy in hypertensive patients, that in itself is independent risk factor for cardiovascular morbidity and mortality [48]. In a cross-sectional study including 4938 subjects from China, the ones with impaired glucose tolerance and newly diagnosed diabetes, but not the isolated impaired fasting glucose, had higher brachial-ankle pulse-wave velocity and thus greater arterial stiffness [49]. In a cross-sectional study of 584 newly diagnosed hypertensive individuals, those who had normal glucose tolerance on 2 hPG but had 1 hPG ≥155 mg/dl had higher indices of vascular stiffness (pulse wave velocity, augmentation pressure, and augmentation index) that correlate with the cardiovascular risk profile [50].

### 3.2. Molecular Studies Relating Postprandial Effect on the Cardiovascular Profile

An experimental study showed that the concentration of soluble forms of the adhesion molecules sE-selectin and sVCAM-1 (vascular cell adhesion molecule-1), which were hypothesized as early predictors of coronary artery disease, was significantly related to postload glucose concentration in 78 men with symptoms of angina and positive exercise stress test who presented for coronary arteriography [51]. In a large observational study, it was found that the oral glucose tolerance test increases biomarkers of systemic inflammation such as hsCRP, IL-6, TNF-alpha, sICAM-1, sVCAM-1, and sE-selectin [52]. Postchallenge hyperglycemia is associated with increased generation of asymmetric dimethylarginine and reactive oxygen species that lead to decreased nitric oxide synthesis which decreases endothelial-dependent flow-mediated dilation and hence decreased vascular function [53]. In a CODAM (Cohort on Diabetes and Atherosclerosis Maastricht) study, *α*-dicarbonyl concentrations are correlated with glucose levels during OGTT, not with FBG or HbA1c in individuals with impaired glucose metabolism and type 2 diabetes which are associated with more vascular complications [54].

### 3.3. Clustering of Cardiovascular Risk Factors with Elevated Postload Glucose

A cross-sectional study of 1475 subjects of Arian ethnicity showed that cardiovascular risk factors like age, body mass index, waist circumference, blood pressure, and lipid profile (not low-HDL cholesterol) were significantly higher in normal glucose tolerance subjects with 1 hPG >155 mg/dl [55]. In a multicenter cross-sectional GENFIEV (GENetics, PHYsiopathology, and EVolution of Type 2 diabetes) study, 1 hPG >155 mg/dl showed lower insulin sensitivity, impaired *β*-cell function, and worse cardiovascular risk profile (glycosylated hemoglobin, blood pressure, low-density lipoprotein cholesterol, and triglyceride) [56]. In the 2005–2014 National Health and Nutrition examination survey, a cross-sectional survey of 3644 adults with prediabetes based on FBG and HbA1c, 6.9% were detected to have diabetes based on 2 hPG and were more likely to have hypertension, higher triglycerides, lower high-density lipoprotein cholesterol, and higher albuminuria [57]. In a DICAMANO study of 447 overweight/obese subjects with FBG ≤5.5 mmol/l (99 mg/dl) and BMI ≥ 25 kg/m^2^ who underwent a 75 g OGTT after multivariable adjustment for FBG, smoking, and physical activity level, the odds ratio (95% confidence intervals) was higher for the presence of postprandial hyperglycemia for anthropometric indices of central fat distribution (neck circumference, waist circumference, and waist-to-height ratio) [58].

### 3.4. Postprandial Glucose Helps to Predict Cardiovascular Disease in Patients with Prediabetes

There has been substantial evidence found by Bergman et al. that led them to support the idea of redefining current diagnostic criteria for prediabetes with elevated 1 hPG level. Among patients having normal glucose tolerance during the OGTT, 1 hPG was found to be highly predictive for detecting progression to diabetes and micro- and macrovascular complications [59].

As compared with subjects with 1 hPG <155 mg/dl, individuals with 1 hPG ≥155 mg/dl exhibited a significantly worse cardiometabolic profile, both in the group without diabetes (HbA1c <5.7%) and in the group with prediabetes (HbA1c 5.7–6.4%) [60]. In the Finnish Diabetes Prevention prospective cohort study, when 504 individuals with IGT were followed up over 13 years with yearly evaluations with OGTT, FPG, and HbA1c levels, it was found that 2 hPG was associated with increased risk of CVD. This supports the use of 2 hPG in screening for prediabetes and monitoring glycaemic levels of people with prediabetes [32]. Both IFG and IGT are associated with increased cardiovascular risk as assessed by serum lipid and hsCRP levels but IGT being characterized by a more atherogenic risk profile than IFG [61]. In the EpiDREAM study, the patients from 21 countries who had IGT and IFG levels were followed up over 3.5 years, and 2 hPG was associated with increased risk of cardiovascular events [22]. In a Chinese cross-sectional study involving prediabetic subjects, odds of developing the CVD events were associated with IGT levels rather than IFG levels [30].

## 4. Conclusion

The concept of relating the postprandial glucose as opposed to fasting glucose to coronary and cardiovascular events began as early as 1970s. This later on formed the basis of large-scale prospective studies that showed that OGTT identifies a largest number of previously undiagnosed diabetic patients with established coronary artery disease. Postprandial glucose was found to be significantly related to myocardial infarction-related mortality in nondiabetic and prediabetic patients. Additionally, studies demonstrated that controlling the postprandial hyperglycemia can prevent cardiovascular disease even in nondiabetic subjects. Biochemical markers of vascular inflammation which were hypothesized as early predictors of coronary artery disease were significantly related to postload glucose concentrations. Cardiovascular risk factors like age, body mass index, waist circumference, blood pressure, and lipid profile were significantly higher in patients with elevated postprandial glucose. The review of these studies suggests the need for reconsideration of factors on the basis of which diabetes is managed in the primary-care clinics. The patients visiting the doctor's office with their fingerstick log are usually uncertain whether they should check fasting sugars or postprandial. Some of these patients are even unsure about the duration between their meals and checking the sugar. They should be counseled about the importance of each of these blood glucose values (fasting vs. one-hour and two-hour postload glucose) and elevation of which of those values is more detrimental for their cardiovascular health. There has also been suggestion by few authors regarding revising the diagnostic criteria for prediabetes based solely on postprandial glucose for early avoidance of risk factors leading to significant cardiovascular morbidity and mortality.

## Figures and Tables

**Table 1:** Epidemiological studies describing the postprandial hyperglycemia as a predictor of cardiovascular mortality in various parts of the world for the last two decades.

No.	Type of study	Name of the study	Total number of patients	Duration of follow-up (years)	Subject characteristics	Study outcome measured	Rate ratio	95% CI	*p* value
1	Prospective cohort	Sievers et al., 1999 [11]	1745	10.6	Pima Indians with type 2 diabetes ≥15 years of age	2-hour postprandial glucose (2 hPG) levels were associated with death rate from cardiovascular disease (CVD)	Death rate 1.2	1.1–1.4	0.007

2	Prospective cohort	de Vegt et al., 1999 [12]	2363	8	Dutch subjects 50–75 years without known diabetes	Postload glucose predictive of increased cardiovascular mortality even within the nondiabetic range	Relative risk (RR) 3.4	1.35–8.53	<0.05

3	Prospective cohort	Meigs et al., 2002 [13]	3370	4	Subjects from the Framingham offspring study without clinical CVD or medication-treated diabetes	2 hPG is associated with cardiovascular events	RR 1.14	1.02–1.27	Not available

4	Prospective cohort	Brunner et al., 2006 [14]	17869	33	London-based male civil servants aged 40–64 years excluding those with known diabetes and with missing glucose measurements	2 hPG associated with coronary heart disease	Hazard ratio (HR) 3.62	2.34–5.56	Not available

5	Prospective cohort	Meisinger et al., 2006 [15]	1160	30	Randomly selected 40–59 year non-diabetic German subjects	1-hour postload glucose (1 hPG) associated with all-cause mortality	HR 1.49	1.17–1.88	Not available

6	Prospective cohort (1974–1979)	Nigam et al., 2007 [16]	1691	14.7	Patients with coronary artery disease (CAD) who were enrolled at 15 centers throughout North America	Postprandial hyperglycemia was not associated with cardiovascular mortality in patients with undiagnosed diabetes	HR 0.89	0.59–1.36	Not available

7	Prospective cohort	Chien et al., 2008 [17]	2165	10.5	Chinese subjects in Taiwan aged ≥35 years	Postchallenge glucose was associated with major cardiovascular events	RR 2.05	1.23–3.42	≤0.001

8	Prospective cohort	Sarwar et al., 2010 [18]	18569	23.5	Iceland subjects without history of diabetes and myocardial infarction (MI)	Postload glucose associated with coronary heart disease	HR 1.03	1.01–1.05	Not available
9	Meta-analysis of 26 western prospective cohort studies	Sarwar et al., 2010 [18]	12652	Not applicable	Not applicable	Postload glucose associated with coronary heart disease	RR 1.05	1.03–1.07	Not available

10	Prospective cohort	Kitada et al., 2010 [19]	422	2	Acute MI (AMI) Japanese patients	2 hPG was the only independent predictor of long-term major adverse cardiovascular events (MACE) two years after AMI	Odds ratio (OR) 1.85	1.07–3.21	0.028

11	Case control	Shimabukuro et al., 2011 [20]	287	Not applicable	Japanese who visited the university hospital to be checked for glucose intolerance or known type 2 diabetes were consecutively recruited	Left ventricle dysfunction associated with impaired glucose tolerance	OR 3.43	1.09–11.2	0.037

12	Prospective cohort (HEART2D trial)	Raz et al., 2011 [21]	1115	2.7	Patients with type 2 diabetes who survived of AMI	Patients using insulin targeting the postprandial versus fasting hyperglycemia had lower cardiovascular events	HR 0.69	0.49–0.96	0.029

13	Large prospective cohort (EpiDREAM study)	Anand et al., 2012 [22]	18,990	3.5	30–85 years multiethnic patients from 21 countries who had impaired glucose tolerance (IGT) and impaired fasting glucose (IFG) levels	2-hour post-OGTT glucose associated with increase in risk of cardiovascular events or death	HR 1.17	1.13–1.22	Not available

14	Prospective cohort	Tamita et al., 2012 [23]	275	5.3	Japanese subjects with AMI	Abnormal glucose tolerance associated with MACE	HR 2.65	1.37–5.15	0.004

15	Prospective cohort	Furtado de Souza et al., 2012 [24]	148	36 ± 14 months	Brazilian subjects undergoing diabetes screening attending a primary care unit	2-hour OGTT results were associated with CVD	OR 1.013	1.002–1.025	0.024
16	9 Finnish and Swedish prospective cohort	Ning et al., 2012 [25]	3743 men and 3916 women	16.4	25 to 90 years who had fasting plasma glucose (FPG) < 6.1 mmol/l and 2 h PG < 7.8 mmol/l and free of CVD	2 hPG associated with coronary heart disease	HR 1.13 in men; 1.33 in women	0.93–1.37 in men; 0.83–2.13 in women	Not available

17	Prospective cohort	Henareh and Agewall, 2012 [26]	123	6.03 ± 1.36	Swedish subjects aged 31–80 years who had suffered a previous MI	2 hPG was a significant predictor of cardiovascular death, recurrent MI, and unstable angina pectoris	HR 1.27	1.00–1.62	<0.05

18	Prospective cohort	Silbernagel et al., 2012 [27]	1772	7.7 ± 2.0	German nondiabetic subjects who were referred for angiography and whose FPG was <126 mg/dl underwent OGTT	Postchallenge glucose undetected by fasting glucose and glycated hemoglobin independently predicted the cardiovascular mortality	HR 1.57	1.02–2.43	0.041

19	Cross sectional (second strong heart study)	Capaldo et al., 2013 [28]	562	Not applicable	American nondiabetic and nonhypertensive Indians of 45–74 years of age	Both higher IFG and IGT levels rather than only IFG associated with left ventricular hypertrophy	OR 9.76	2.03–46.79	0.004

20	Prospective cohort	Barzin et al., 2013 [29]	3794	8	Tehran urban subjects aged ≥40 years without history of diabetes or CVD	Isolated postchallenge hyperglycemia associated with cardiovascular events	HR 1.77	1.19–2.64	0.005

21	Cross sectional	Yang et al., 2013 [30]	6040	Not applicable	Chinese prediabetic subjects	CVD events associated with IGT levels compared to IFG levels	OR 2.88	1.36–6.01	0.0059

22	Prospective cohort	Kuramitsu et al., 2013 [31]	828	4.3	Japanese patients of stable angina undergoing percutaneous intervention (PCI)	Postchallenge hyperglycemia was associated with MACE	HR 1.62	1.07–2.53	0.023

23	Finnish diabetes prevention prospective cohort study	Lind et al., 2014 [32]	504	13	Finnish individuals with IGT were followed up with yearly OGTT, FPG, and HbA1c	2 hPG was associated with CVD events	HR 2.19	1.51–3.18	≤0.001
24	Prospective cohort	Ritsinger et al., 2015 [33]	167 AMI patients and 184 controls	10	Swedish patients up to 80 years with AMI (*n* = 167) and healthy controls (*n* = 184) with no previously known diabetes	Patient with AMI having abnormal glucose tolerance after an OGTT performed at the time of discharge had higher cardiovascular mortality	HR 2.3	1.24–4.25	0.008

25a	Yorkshire retrospective cohort	George et al., 2015 [34]	768	3	Patients without pre-existing diabetes mellitus post-MI	IGT associated with increased incidence of MACE	HR 1.54	1.06–2.24	0.024

25b	Yorkshire retrospective cohort study	George et al., 2015 [34]	768	3	Patients without pre-existing diabetes mellitus post-MI	Newly diagnosed diabetes associated with increased incidence of MACE	HR 2.15	1.42–3.24	0.003

26	Prospective cohort	Faghihi-Kashani et al., 2016 [35]	2607	7.2	Patients of type 2 diabetes mellitus in Tehran	2 hPG was associated with high incidence of coronary heart disease	HR 1.64	1.03–2.61	Not available

27	Prospective cohort	Shahim et al., 2017 [36]	4004	2	24 European subjects aged ≥18–80 years hospitalized for a first or recurrent CAD event	2 hPG associated with cardiovascular events	HR 1.38	1.07–1.78	0.01

28	Prospective cohort	Nielsen et al., 2017 [37]	4934	27	Swedish subjects without diabetes	1 hPG predicted the cardiovascular death	HR 1.09	1.01–1.17	0.02

29	Prospective cohort	Chattopadhyay et al., February 2018 [38]	674	4	Post-MI survivors without known diabetes in England and Wales	Only 2 hPG predicted MACE	HR 1.12	1.04–1.20	≤0.001

30	Retrospective cohort	Chattopadhyay et al., August 2018 [39]	1056	40.8 months	Acute coronary event survivors without known diabetes mellitus who had FBG and 2 hPG measured predischarge	2 hPG independently predicted MACE	HR 1.091	1.043–1.142	≤0.001

31	Cross sectional (CATAMERI study)	Fiorentino et al., 2019 [40]	1010	Not applicable	Nondiabetic Caucasian individuals with hbA1c <5.7%	1 hPG during OGTT ≥ 155 mg/dl associated with CAD	OR 6.16	1.05–36.32	0.04

32	Retrospective cohort	Chattopadhyay et al., 2019 [41]	1056	2.8	MI survivors in East yorkshire and North Lincolnshire	2 hPG predicted MACE-free survival	HR 1.16	1.07–1.26	≤0.001
